# Toothache of Horses

**Published:** 1887-04

**Authors:** T. S. McCart


### TOOTHACHE OF HORSES.

T. S. McCart, V. S.

It seems to me that a short talk about the care and feeding of horses might be beneficial to many lovers and owners of horses. Who ever thinks of his horse having toothache, or that his horse's mouth needs attention, or that there is anything wrong with his horse's mouth when he throws off large quantities of saliva? Continually chewing the bit, shaking the head, lopping the ear, turning the head sideways, and a score of other unnatural motions the animal makes to let you know that he is suffering pain. A horse cannot thrive well with a decayed tooth continually annoying him. He will not masticate his food properly because it causes such intense pain to bring the tender tooth in contact with any substance, however soft; consequently the animal will bolt sufficient feed to appease his hunger, and nothing more. After a time he grows thin in flesh, the hair becomes rough and staring, he is easily fatigued, has no ambition to move, etc. He now becomes a subject for physic, condition powders, etc. Finally some learned horse doctor undertakes the case and warrants to cure it in a week. After a few days the doctor asks for more time, as it is more difficult than he expected. The outcome of the matter is, the owner gets a bill three times the value of the horse, with the advice to turn the animal to grass for the season. Of course, he will not improve as long as the cause of the difficulty remains, and is at last pronounced worthless, destroyed, or sold for a trifling sum.

All of this annoyance and loss would have been obviated by timely exercise of a little good horse-sense! When your horse does not thrive, you can be certain there is a cause for it, which you can find by giving a little attention to the matter.—*Minnesota Medical Monthly.*

